# Considering the trade-offs of environmental, social, and governance risk in digital health

**DOI:** 10.3389/fdgth.2026.1947743

**Published:** 2026-10-28

**Authors:** Yacine Hadjiat, David D. McManus, Carlos Montana, Yih Yng Ng, Leo A. Rezayan, Lisa Scharoun, Yoan Sebastien Sapanel, Anna Berbenyuk

**Affiliations:** 1 Dubai Health Innovations, Mohammed Bin Rashid University of Medicine and Health Sciences, College of Medicine, Dubai Healthcare City, Dubai, United Arab Emirates; 2 French National Institute of Health and Medical Research (INSERM) U987, Boulogne-Billancourt, France; Paris Cité University, Paris, France; 3 Department of Medicine, University of Massachusetts Chan Medical School and UMass Memorial Health, Worcester, MA, United States; 4 Interdisciplinary Arts and Design Department, Rochester Institute of Technology, Dubai, United Arab Emirates; 5 Saw Swee Hock School of Public Health, National University of Singapore, Singapore, Singapore; 6 Centre for Healthcare Innovation, Singapore, Singapore; 7 School of Design, Queensland University of Technology, Brisbane, QLD, Australia; 8 Design School, Taylor's University, Subang Jaya, Malaysia; 9 Faculty of Creative Industries, Education and Social Justice, Queensland University of Technology, Brisbane, QLD, Australia; 10 Yong Loo Lin School of Medicine, Institute for Digital Medicine, National University of Singapore, Singapore, Singapore

**Keywords:** artificial intelligence, carbon footprint, digital health, digital medicine, eHealth, environment social governance (ESG), healthcare inequality, sustainable development

## Abstract

**Introduction:**

Digital health innovations are increasingly recognized as cost-efficient and scalable solutions to improve healthcare delivery and accelerate progress toward the Sustainable Development Goals (SDGs). However, limited research has examined these technologies within broader socio-economic and sustainability contexts, particularly regarding Environmental, Social, and Governance (ESG) risks and potential trade-offs.

**Methods:**

This narrative review integrates digital health within an environmental, social, and governance (ESG) framework. Literature on digital health, sustainability, and ESG-relevant risk was identified through searches of PubMed/MEDLINE, Scopus, Web of Science, and Google Scholar, supplemented by reference-list screening and purposive inclusion of policy and institutional reports; searches were last updated in May 2026. Eligible sources addressed digital health technologies and engaged with at least one ESG domain. Sources were synthesised thematically across the environmental, social, and governance domains, and a conceptual framework was developed to support evaluation of ESG-related trade-offs in digital health implementation**.**

**Results:**

The review highlights that digital health solutions, while offering significant benefits in accessibility and efficiency, may exacerbate health inequalities, increase environmental burdens through energy consumption and carbon emissions, and raise governance concerns related to ethics, data privacy, and regulation. The findings emphasize the interdependence of ESG factors and demonstrate that isolated evaluation of these dimensions can lead to incomplete or misleading conclusions. A preliminary framework is proposed to guide the systematic assessment of ESG trade-offs in digital health interventions.

**Discussion:**

Integrating ESG considerations into digital health innovation is essential to ensure sustainable and equitable healthcare outcomes. The proposed framework provides a foundation for evaluating the broader impacts of digital health technologies, though further research is needed to develop standardized metrics and a comprehensive ESG taxonomy. Incorporating ESG principles into policy and implementation strategies will enhance the long-term effectiveness and societal value of digital health solutions.

## Introduction

Digital health is a fast-developing field that can be defined as “systems, tools, and services based on information and communications technology that can be used to diagnose, monitor, and treat patients, as well as to collect and share a patient's health information” (1). Its emergence coincides with a broader paradigm shift in health, in which care is moving from episodic, facility-based and largely reactive delivery towards models that are continuous, distributed, data-driven, and oriented towards prediction and prevention across both clinical and population health (2–5). Within this shift, digital health solutions have moved beyond internet-linked applications to encompass advanced analysis and interpretation, including genomics, artificial intelligence, wearables, digital biomarkers, and telemedicine (2, 4, 6, 7). The World Health Organization's global strategy on digital health positions these technologies as instruments of health system strengthening rather than as discrete products, and frames their value in terms of equitable access, quality, and resilience (5). Consistent with this framing, digital health innovations are often presented as mechanisms to reach underserved or unserved populations, as well as a means of connecting clinicians and patients in a data-driven and empowering manner (3, 4, 6). While the health and social benefits of digital solutions present significant opportunities, the full environmental, social, and governance (ESG) risks associated with these solutions should be considered when implementing them across all contexts. As with all ESG considerations, the environmental, social, and governance factors are interrelated and influence one another to varying degrees. The implementation of any digital health innovation must consider not only the ESG-related risks and opportunities, but also their integration and combined impact. Evaluating each risk in isolation may lead to an incomplete understanding or to an inadequate consideration of trade-offs among different ESG factors.

The ESG construct originated outside health, in sustainable finance and corporate accountability, as a means of appraising organizational performance against non-financial dimensions that nonetheless pose material risks. The term was consolidated in the 2004 Who Cares Wins report convened under the United Nations Global Compact, which argued that environmental, social, and governance factors are financially material rather than peripheral. It was subsequently institutionalised through the Principles for Responsible Investment and, more recently, through mandatory sustainability disclosure standards (8–10). Three features of the construct make it useful as an analytical lens for digital health. First, it is explicitly multidimensional, requiring environmental burden, social distribution, and institutional accountability to be appraised together rather than sequentially. Second, it concerns externalities — costs and benefits accruing to parties other than the purchaser or the immediate beneficiary — which is precisely the analytical problem posed by digital health technologies whose energy, materials, and labor inputs are geographically remote from the patients who benefit from them. Third, it is decision-oriented, having been developed to inform procurement, investment, and disclosure rather than to describe phenomena. Health technology assessment has begun to incorporate environmental criteria along comparable lines (11), and adjacent work on digital health equity has argued that sustainability should be treated as a design parameter rather than as an afterthought (12, 13). ESG is therefore adopted here as the organising framework for this review rather than as an object of study in its own right: it supplies the categories through which the digital health literature is interrogated.

A rising concern with digital solutions is the materials used in their production (e.g., metals and hardware), as well as their energy use and dependence. The use of these innovations is increasingly being questioned considering their environmental impact, particularly in the context of AI and computing power (11). While these concerns are important, outright rejection of digital solutions on this basis may overlook their substantial social and health benefits. It is therefore essential that digital solution implementation and feasibility planning explicitly and rigorously incorporate broader sustainability and ESG-related risks and opportunities considerations. A comprehensive assessment of ESG-related risks and opportunities should be undertaken for all innovations, regardless of their environmental benefits. In the context of digital health solutions, a potential starting framework for such considerations is presented.

Despite the growing adoption of digital health technologies, limited research has systematically examined these innovations through an integrated ESG lens, particularly regarding trade-offs among sustainability dimensions. This review therefore has three aims: to characterize the environmental, social, and governance risks and opportunities reported in the digital health literature; to identify where gains in one ESG domain are achieved at cost to another; and to propose a conceptual framework through which such trade-offs can be made explicit in the design, procurement, and evaluation of digital health interventions.

## Methods

### Review design and scope

This study adopts a narrative review approach to examine digital health innovations through the lens of ESG considerations. Given the emerging, interdisciplinary, and context-dependent nature of ESG in digital health, a narrative methodology was selected to enable a critical and integrative exploration of risks, opportunities, and trade-offs. The purpose of this review was to situate digital health solutions within broader socio-economic and environmental systems and to explore how these innovations may simultaneously contribute to sustainability goals. In this regard, the review explicitly focuses on the interconnected nature of ESG factors and the need to consider these dimensions collectively when evaluating digital health interventions.

The scope of the review included a wide range of digital health technologies, including AI, digital therapeutics, telemedicine, wearable technologies, and data-driven health systems. These technologies are examined in relation to:

• Environmental considerations, such as energy consumption, carbon emissions, and material resource use

• Social considerations, including equity, access, inclusion, and digital capability

• Governance considerations, including ethics, regulation, data privacy, and accountability

### Conceptual framework

ESG functions in this review as the analytical framework through which the literature was interrogated, rather than as the phenomenon under investigation. The three domains were specified *a priori* as the organising categories for data extraction and synthesis, on the grounds set out in the Introduction: their multidimensionality, their orientation towards externalities, and their decision-oriented origins. Two consequences follow for the methodology. First, the domains and their sub-dimensions were used as an extraction matrix, so that literature addressing — for example — data-centre energy demand, digital exclusion, or algorithmic accountability was assigned to a domain irrespective of whether the source itself employed ESG terminology. Second, ESG terminology was deliberately not used as a search term because the vast majority of relevant digital health literature does not adopt the vocabulary of sustainable finance; searching for it would have restricted retrieval to a small and unrepresentative subset of the field. The search was instead constructed around the substantive concepts that populate each domain.

### Eligibility criteria

Sources were eligible if they addressed digital health technologies — including artificial intelligence applied to health, digital therapeutics, telemedicine, wearable and remote monitoring technologies, or health data infrastructure — and engaged with at least one of the three ESG domains as defined above. Both empirical studies and conceptual or framework papers were eligible. Grey literature was included where it contributed evidence not available in the peer-reviewed record, specifically national digital health strategies, intergovernmental and institutional reports, and technical energy-sector analyses. Sources were excluded if they addressed digital technologies without a health application, addressed health system sustainability purely in financial terms without environmental, social, or governance content, or were not available in English. No restrictions were placed on the study design, and no formal appraisal of methodological quality was undertaken, consistent with the interpretive aims of a narrative review.

### Search strategy

Literature was identified through iterative searching of PubMed/MEDLINE, Scopus, Web of Science, and Google Scholar. Searches were first conducted in August 2024 and were last updated in May 2026. Consistent with a narrative rather than a systematic design, the search was exploratory and developed as the review progressed, with terms added as concepts emerged during reading. It was intended to capture both technical and conceptual literature, reflecting the topic's multidisciplinary nature, rather than to achieve exhaustive retrieval.

Keywords and search terms included combinations of:

- “Digital health”, “eHealth”, and “digital medicine”
- “Artificial intelligence” AND “healthcare”
- “Health equity”, “digital divide”, and “access”
- “sustainability”, “carbon footprint”, and “energy use”
- “Data governance”, “algorithmic accountability”, “AI regulation”, and “health data privacy”

Boolean operators (AND, OR) were applied to refine search results. In addition, the reference lists of relevant articles and reviews were manually screened to identify additional sources. Policy documents and institutional reports were also included where they contributed to understanding ESG considerations in real-world implementation contexts.

This review is an interpretive narrative synthesis and is reported as such. Sources were selected purposively, prioritising conceptual contribution and coverage across the three domains rather than exhaustiveness; records were not screened sequentially against pre-specified criteria, and screening counts were not maintained. A PRISMA-style flow diagram is accordingly not reported, since the reporting conventions of systematic and scoping reviews do not correspond to the method used here. The implications for reproducibility are addressed in the Limitations section.

Data from included sources were extracted and organized according to the three ESG domains, while recognizing their interdependence and overlap:

- Environmental factors: energy demand, carbon footprint, hardware lifecycle, and resource extraction
- Social factors: access, equity, inclusion, digital literacy, and socio-economic determinants
- Governance factors: ethics, regulation, data governance, transparency, and accountability

A thematic synthesis approach was employed to identify recurring patterns, tensions, and gaps across the literature. Attention was given to:

- Trade-offs between ESG dimensions, especially where benefits in one domain may generate risks in another
- Implications for the Sustainable Development Goals (SDGs), particularly the relationship between SDG 3 (health) and other goals such as SDGs 7, 10, 12, and 13
- The extent to which digital health interventions are context-sensitive or risk reinforcing existing inequalities

### Framework development

Building on the thematic synthesis, this study proposed a conceptual ESG-informed framework for evaluating digital health innovations. The framework was developed iteratively to reflect the central argument of this review: that decision-making in digital health should account for the integrated and often competing demands of environmental, social, and governance factors.

The development process involved:

1. Identifying key ESG-related variables and considerations emerging from the literature
2. Examining how these factors interact, overlap, and generate trade-offs in real-world contexts
3. Structuring these relationships into a decision-oriented framework to support the evaluation, design, and implementation of digital health interventions

## Results

The synthesis is organised below by ESG domain. Table 1 summarises the themes identified within each domain, the principal sources contributing to each, the trade-offs that emerged across domains, and how each theme informed the conceptual framework presented in the Discussion.

**Table 1:** Thematic synthesis across the three ESG domains, showing themes identified, contributing sources, cross-domain trade-offs, and the corresponding framework element.

ESG domain	Theme identified	Principal contributing sources	Cross-domain trade-off identified	Corresponding framework element
Environmental	Energy demand of computation and data centres	Thompson (25); Gordon et al. (26); Asongu et al. (27); Dwivedi et al. (28); IEA (22); Spencer & Singh (40)	Health gains under SDG 3 are obtained against energy and emissions burdens governed by SDGs 7 and 13; per-unit efficiency gains may be offset by expanded deployment	Environmental level: energy source and carbon intensity of compute
Environmental	Hardware lifecycle, mineral extraction and e-waste	Turley et al. (31); Jungblut (23); Pan et al. (22); Almulhim et al. (23)	Device-dependent access, a social benefit, presupposes extraction and disposal burdens borne disproportionately by low-resource settings	Environmental level: resource availability, scarcity, circular economy
Environmental	Net environmental effect is deployment-specific, not intrinsic to the technology	Lokmic-Tomkins et al. (36); Reynolds (37); Purohit et al. (44)	Telemedicine may reduce travel emissions while adding computational burden; the direction of the net effect is unresolved and rarely quantified	Environmental level: context-specific impact assessment rather than technology-class judgement
Social	Digital exclusion and eligibility barriers	Hadjiat (6); Sapanel et al. (15); Lakhotia et al. (3); Kettle (19)	Interventions that expand access in aggregate may widen relative inequity, setting SDG 3 against SDG 10	Social level: access, digital literacy, socio-economic determinants
Social	Equity as a design parameter rather than an outcome measure	Bucher et al. (18); Crawford & Serhal (20); Crawford et al. (21); Kim & Backonja (45)	Equity-focused design raises development cost and may slow deployment, deferring benefit to reach it more evenly	Social level: inclusive design, bias mitigation, continuous evaluation of access barriers
Social	Under-representation of and dependency in low- and middle-income settings	Lakhotia et al. (3); Pan et al. (22); Almulhim et al. (23); Generation Climate Europe (24); Coetzer et al. (13); Tangcharoensathien et al. (4)	Benefits accrue predominantly to high-income settings while environmental and labour burdens fall elsewhere; externally supplied capability makes continuity of care contingent on arrangements outside the health system	Social–environmental interface: geography and location as a modifier of both benefit and burden
Social	National inclusion strategies as social policy instruments	Australian Digital Health Agency (16); Centre for Public Impact (9)	State-level access guarantees depend on governance and fiscal capacity that many systems lack, so the instrument is least available where the need is greatest	Social–governance interface: policy instruments for access and inclusion
Governance	Data stewardship: ownership, consent, secondary use, cross-border transfer	Dwivedi et al. (28); Coetzer et al. (13)	The infrastructure enabling population-level insight is the same infrastructure enabling surveillance and commercial exploitation of health data	Governance level: data sharing, privacy, ownership
Governance	Algorithmic accountability, bias and opacity	Bucher et al. (18); Kim & Backonja (45)	Model performance may improve with data scale while inspectability declines, making responsibility for a clinical decision harder to locate	Governance level: AI bias, transparency, accountability
Governance	Regulatory and procurement architecture	Alami et al. (11); Thompson (25); Jungblut (23)	Procurement can enforce environmental criteria but adds transaction cost and may narrow the supplier pool, with implications for access and price	Governance level: procurement criteria and health technology assessment
Governance	Policy coherence across health, digital and environmental mandates	Tangcharoensathien et al. (4); WHO (5)	Separate institutional mandates leave inter-domain trade-offs unadjudicated, which is the mechanism through which environmental and social risks go unaddressed	Governance level: alignment of health and environmental policy

Reference numbers correspond to the reference list in the main manuscript. Framework elements map to Figures 1 and 2. Themes assigned to an interface between two domains are those in which the trade-off could not be located within a single domain without loss of meaning.

### Digital health implementation towards sustainability

Digital health interventions have been widely positioned as enablers of Sustainable Development Goal 3 (SDG 3), which aims to ensure healthy lives and promote well-being for all. These technologies expand access to healthcare, improve system efficiency, and enable data-driven decision-making. However, their contribution to sustainability extends beyond health alone and intersects with other SDGs, including SDG 7 (Affordable and Clean Energy), SDG 10 (Reduced Inequalities), SDG 12 (Responsible Consumption and Production), and SDG 13 (Climate Action) (14). Digital health solutions have been positioned as a means of expanding the reach of healthcare provision (1); however, concerns have been raised that they may exacerbate inequality rather than address it (15). For example, Sapanel et al., in a review of digital therapeutics and their economic evaluation, found overall economic benefits while raising concerns about methodology and about the capacity of these interventions to enable access (15). Biases in participant recruitment illustrate the difficulty: in 11 of the 18 studies reviewed, participants were required to have internet access, a smartphone or tablet, the skills necessary to use a personal computer, medical insurance, or employment to take part. Such requirements may limit the participation of members of marginalized or socioeconomically vulnerable groups, and only 3 studies addressed the ethical and distributional issues inherent in implementing digital technologies (15). Lakhotia et al. observe that although access to mobile phones exceeds access to sanitation in many settings, the inclusiveness of that access remains a concern; digital health interventions offer a means of reaching more people, supporting overburdened healthcare systems, improving record-keeping, and enabling AI-based analysis of large datasets (3). Australia's National Digital Health Strategy 2023–2028 illustrates how governments are developing frameworks to secure broad access to digital health, setting out a plan to improve access to and use of digital health services across all population groups, regardless of socioeconomic status, disability, or other potential barriers (16). Singapore's digital inclusion initiatives provide a further example (17). Bucher et al. highlight the necessity of incorporating equity dimensions into the design of digital health tools, and social and ESG considerations have been incorporated into frameworks for evaluating the efficacy of digital health implementation (18), recognizing that such technologies are not socially neutral.

### Social risks: equity, access, and inclusion

Although digital health solutions increase healthcare access and, in turn, improve health outcomes, the possibility that these innovations may worsen existing health inequities must be meticulously considered. Due to the need for internet access, digital competence, and compatible devices, socioeconomically vulnerable groups may be systemically excluded from accessing digital interventions (6, 15, 19). A study by Sapanel et al. found that most digital therapeutic studies required participants to have internet access, a smartphone, or insurance, thereby limiting the inclusion of disadvantaged populations (15). These requirements risk exacerbating health disparities, particularly in settings where access to technology is scarce (3, 6).

Integrating equity dimensions early in the design and evolution of digital health solutions is essential to ensuring these interventions meaningfully contribute to SDG 3 (“Ensure healthy lives and promote well-being for all at all ages”). Examples include the Digital Health Equity Framework (20, 21) and the equity-focused design strategies reviewed by Bucher et al. (18). These frameworks integrate social and governance considerations into digital health programs and emphasize inclusive recruitment, bias mitigation in AI models, and ongoing assessment of access barriers.

To address these challenges, equity-focused frameworks emphasize inclusive design, bias mitigation in AI, and continuous evaluation of access barriers. These approaches highlight the importance of integrating social considerations early in the design and implementation of digital health interventions (7). For these tools to be utilized equitably in real-world settings without causing harm, strict ethical health policies and procurement practices must be rigorously implemented, including the preservation of data privacy and algorithmic transparency.

### Perspectives from low- and middle-income countries

The evidence identified in this review is drawn predominantly from high-income settings, and this imbalance itself constitutes a social risk: where digital health is positioned as a route to universal health coverage, the settings with the largest coverage gaps are those least represented in the evidence base on which implementation decisions are made. The relevant considerations differ in both kind and degree. Connectivity and device access are more constrained and more strongly gendered; the digital divide frequently follows existing lines of rural residence, income, language, and literacy; and health systems may lack the data infrastructure, workforce capacity, and regulatory apparatus that digital interventions presuppose (3, 4). Lakhotia et al. note that mobile phone access exceeds access to sanitation in many settings, but caution that it is the inclusiveness of that access, rather than its extent, that determines health benefit (3). A distinctive dependency risk also arises: when external vendors or donors supply digital health capabilities, continuity of care becomes contingent on arrangements over which the health system has limited control, and health data may be governed by terms set elsewhere (4, 22). At the same time, low- and middle-income countries bear a disproportionate share of the environmental costs of digital infrastructure through mineral extraction and e-waste while receiving fewer of its benefits (22–24) — the clearest instance in this review of an ESG trade-off in which environmental, social, and governance burdens fall on one population while the benefits accrue to another. Because narrative design permits engagement with contexts not represented in the retrieved empirical literature, this asymmetry is discussed explicitly here; it should not, however, be read as a synthesis of evidence from these settings, which remains sparse.

### Environmental impacts of digital health

Digital health technologies are associated with substantial environmental costs. These include energy consumption by data centres, carbon emissions from computational processes, and environmental degradation linked to hardware production and disposal (25, 26). The global transition to clean energy remains slow, yet the energy requirements of digital solutions, particularly those based on AI, are high. Although these requirements may fall over time, the need for sustainable energy remains critical. Many nations continue to rely on fossil fuels for electricity generation, and electricity generation therefore remains a major driver of climate change (27). Digital technologies are not carbon neutral and can incur high energy costs: blockchain, for example, consumes large amounts of energy and is associated with between 22 and 29 million metric tonnes of carbon dioxide emissions annually (28). The 2023 United Nations Conference of the Parties (COP 28) called for accelerated mitigation and adaptation and urged governments to move more decisively away from fossil fuels towards renewable energy (28, 29). These considerations should, and likely will, become integral to public health programs, particularly those that are digitally driven (19). The question that arises is how to balance the imperative to protect and improve health outcomes against the energy requirements of health interventions. Not all health interventions place equal strain on environmental resources, and the environmental impacts experienced by the populations they serve may themselves vary considerably.

At the same time, the social and health consequences of poor environmental outcomes place further strain on healthcare systems. The consideration of ESG in digital health solutions is therefore nuanced and is not often examined in an integrated manner (30). This creates a disproportionate burden on regions that may benefit least from digital health innovations.

### Systemic trade-offs and environmental justice

The environmental costs described above are not distributed in proportion to the health benefits they generate. Digital health interventions advance SDG 3 while drawing on the energy, materials, and waste-absorption capacity governed by SDGs 7, 12, and 13 (5, 17, 18), and the resulting tension is structural rather than incidental: the computational intensity that enables population-scale analysis is the same property that generates the emissions burden (31, 32). Analytically, what follows is that environmental impact cannot be treated as a fixed attribute of a technology, but must be assessed against the health benefit obtained, the population served, and the energy system within which the technology operates.

Low-resource countries with limited environmental safeguards are disproportionately affected by e-waste and by the mining of the minerals required for digital devices (33). These effects can be mitigated through a circular economy approach that emphasises device recyclability, responsible sourcing, and energy labelling for AI (26, 33). If digital health innovations are not to undermine the environmental and social commitments that states have made nationally and globally, they require regulatory frameworks capable of holding those commitments in view (25, 33).

This asymmetry is the substance of environmental justice concern. The life cycle of digital health hardware — from mineral extraction, frequently in low- and middle-income settings, through to disposal as toxic e-waste — transfers environmental harm to the communities that benefit least from the resulting innovations (22, 23, 30). Environmental degradation and climate change in turn damage health directly and increase demand on the health systems least equipped to meet it (22, 30, 34, 35), so that the environmental costs of digital health return as social and clinical costs, borne elsewhere.

These considerations point towards policies that prioritise renewable energy in data centers, support circular economy approaches to device manufacturing, and encourage the development of energy-efficient AI models. Analysis of the competing demands between health innovation and environmental impact is not an ethical supplement to evaluation but a condition of it, if digital health is to promote rather than erode planetary well-being (24, 25, 29, 36–38).

### Governance risks: ethics, regulation, data stewardship, and accountability

Governance was specified as the third extraction domain, and eligible evidence was identified, although this evidence is more fragmented than in the environmental and social domains and is rarely framed in explicitly governance terms. Four recurring concerns can be distinguished.

The first concerns data stewardship. Digital health technologies generate and depend upon continuous personal data, and questions of ownership, consent, secondary use, and cross-border transfer remain unresolved in most implementation contexts (13, 28). Where data governance is weak, the infrastructure that enables population-level insight is the same infrastructure that enables surveillance and the commercial exploitation of health information.

The second concerns algorithmic accountability. Models trained on non-representative data reproduce and may amplify existing disparities, and the equity-focused frameworks identified in this literature consistently treat bias mitigation and transparency as governance obligations rather than as technical preferences (13, 28). Accountability is further complicated by opacity: where a model's reasoning cannot be inspected, responsibility for a clinical decision becomes difficult to locate.

The third concerns regulatory and procurement architecture. Regulatory frameworks for digital health remain uneven across jurisdictions, and environmental criteria are largely absent from health technology assessment and procurement processes, even though procurement is the point at which most digital health technologies enter health systems (11, 25). National strategies such as Australia's provide governance instruments for access and inclusion (16), but comparable instruments for environmental accountability are rare.

The fourth concerns policy coherence. Health policy, digital policy, and environmental policy are typically formulated by separate institutions with separate mandates, so that trade-offs between them are seldom adjudicated explicitly (4, 5). Governance failures of this kind are not incidental to environmental and social risk; they are the mechanism through which such risks go unaddressed.

## Discussion

The discussion below follows the order of the results. It considers, in turn, the social distribution of digital health benefits, the environmental costs of these technologies and the procurement mechanisms through which those costs might be governed, the governance conditions required if either is to be managed, and finally the integration of the three domains into a single decision framework. The argument throughout is that these domains are not separable, and that the practical difficulty facing decision-makers is not the identification of risks within each domain but the adjudication between them.

### SDG 3 (health) interventions must consider integrated ESG factors

Technological interventions for climate change adaptation and mitigation are valuable, but should not be regarded as holistic, comprehensive, or rapid solutions. Pragmatically, it is important to account for the transition from established energy sources to renewables, and for similar initiatives intended to offset climate change impacts (39), which should be evidence-based and developed with integrity (39). The same is true of digital health innovations. As a practical example, the opportunities offered by AI and generative AI models may deliver gains in efficiency, diagnosis, and data management. Yet, the energy implications of the increasingly widespread use of large language models are rarely considered explicitly (40). The cost is borne by the wider system rather than by the immediate beneficiaries. Any health intervention program must therefore consider these factors in the context of the wider public health system, both in terms of its capabilities and its longer-term national and global impacts. This illustrates the need to identify, quantify, and evaluate such costs within health programs, and specifically to articulate the cost when an AI solution is chosen. In some cases, a more energy-efficient solution may be sufficient, eliminating the need for a large language model. Much as food labelling enables consumers to make an informed choice, energy labelling for AI-enabled health technologies would make this trade-off visible at the point of decision.

The point of decision, however, is rarely the patient. Digital health technologies are overwhelmingly procured by hospitals, health systems, insurers, and governments, which means that disclosure will alter behaviour only if it is embedded in the instruments those bodies actually use: tender specifications, health technology assessment criteria, framework agreements, and supplier qualification requirements. Alami et al. found that environmental considerations are largely absent from digital health technology assessment and procurement, notwithstanding stakeholder recognition of their relevance (11). Concretely, public tenders for AI-enabled health technologies could require disclosure of training and inference energy consumption, the carbon intensity of the compute on which the technology depends, hardware lifecycle and take-back arrangements, expected device replacement cycles, and energy efficiency per unit of clinical output. Such disclosures could be weighted within award criteria alongside clinical performance and cost. Procurement is also the stage at which the asymmetry described above can be corrected, since it is the one point at which a health system can decline a technology whose environmental costs are borne by populations other than its own (11, 25, 33, 36).

As with the above, digital healthcare solutions, while offered as significant means of expanding healthcare access for more people, have the potential to entrench unequal health access patterns (2). Healthcare solutions, regardless of complexity, must be designed with available resources in mind. Systemically, digital health innovations must be considered within the regulatory environment (and relevant environmental and SDG commitments the country has made), procurement priorities, economic requirements, professional skillsets, population health needs, and scientific factors (31). This may require balancing potentially environmentally damaging technology against broader public health initiatives – but if so, it should be considered in a rigorous, deliberate, and transparent manner (25, 26, 36).

For example, AI may assist in health innovations (7), yet it is not socially or environmentally neutral. The associated computing and electricity costs of digital technologies are high, and toxic electronic waste is generated at high levels, much of which is shipped to lower-resource areas, potentially exacerbating health disparities (31). Equally, minerals required for technological devices and renewable energy technologies are mined in developing countries, often with fewer health, safety, and human rights requirements (31). Therefore, while digital health innovations may improve health outcomes and advance SDG 3, they are equally likely to undermine other SDGs, such as emission reduction and poverty alleviation. Hence, a decision-making framework is needed to guide procurement, implementation, and evaluation decisions.

The commitment in SDG 3 to ensure healthy lives and promote well-being (14) may be addressed directly through accessible digital solutions, for instance wearable technologies that track and monitor vital signs, or AI software capable of analysing radiological images. Yet these benefits may come at the cost of other SDGs (38). Such innovations may be available only to certain populations, thereby entrenching inequalities (6) and working against SDG 10 (reduced inequalities). Frameworks for evaluating these ESG costs and the balance of impacts are resource-dependent and lack clear evaluation criteria; this remains an area requiring substantial further debate and research. Artificial intelligence applied to medical imaging, for example, has been shown to match or exceed expert performance on defined detection tasks and to shorten reporting turnaround times, which may reduce time to diagnosis and treatment (41–43). Gains of this kind may relieve pressure on healthcare systems and, where access is deliberately designed for, support more equitable provision. The environmental consequences, however, should not be assumed: reductions in patient travel or repeat imaging may offset computational burden in some deployments, but this is an empirical question that depends on the model, deployment, and underlying energy system, and it has rarely been quantified (36, 37, 44).

### An ESG framework for considering digital health innovations

For digital solutions in healthcare and the SDGs, Hadjiat presents an equity framework to evaluate or construct designs (6). This framework may be adapted to incorporate future thinking — in effect, considering the health impacts of climate change and adaptation on countries and communities (34). This is the fuller ESG approach: climate and environmental issues cannot be considered in isolation, because social and governance concerns are themselves drivers of climate change effects, actions, and mitigants (12, 13, 34, 35). Changing temperatures alter disease prevalence, rainfall levels affect water-borne disease, and water scarcity may introduce further disease burdens, each of which bears on a health system's capacity to manage the scope and level of disease in its communities. Foresight is therefore essential: what is predicted, what will be mitigated, and how will this affect people's health? The closure of a fossil fuel energy source may reduce pollution. Still, it could also precipitate an economic downturn, restrict access to alternative energy, increase unemployment, and raise the number of people unable to access preventive healthcare and treatment. Extreme weather events, in turn, carry direct adverse health consequences.

In a review of equity frameworks, Bucher et al. (18) offer approaches to designing inclusive health interventions. Their focus is the design stage, but SDG 3 is inherently concerned with health equity. Equity tools, we argue, offer a useful, ecosystemic view of health interventions and are therefore amenable to adaptation to ESG considerations. As Bucher et al. point out, equity considerations in health interventions are necessary not only to illustrate potential impact, but also to design an intervention that secures buy-in, is implemented, and is sustainable, and is therefore cost-efficient and effective in the long run (12, 18). The authors reviewed four frameworks; while each offers specific detail, their collective utility lies in providing structure for the broader factors bearing on health intervention design. At the ESG level, these frameworks do not always encompass the full range of environmental or governance factors, but they address the social domain thoroughly. Because they focus on inclusion, it is understandable that they do not address the interplay and integration of the environmental, social, and governance dimensions. Notwithstanding this, Bucher et al. encourage attention to socio-economic factors such as race, gender, age, geography, socio-economic status, occupation, and religion, as well as to events that create vulnerability or disadvantage. Importantly, these frameworks consider the interplay of such factors, with several explicitly attending to the levels at which individuals engage: individual, interpersonal, community, and societal (18).

### Interpretation in the context of previous studies

The Digital Health Equity Framework (20) provides a starting point for considering digital health innovations sustainably, balancing positive and negative impacts on the SDGs. Developed in the context of COVID-19, the framework sets out the factors that health interventions must consider during development and implementation, and it was subsequently operationalized as a health equity impact assessment supplement for digital health (21). At its core, the framework concerns trade-offs between competing interests, competing priorities, and policy positions, and could well be extended to encompass competing environmental and social outcomes. It is principle-based, providing a thinking model through which the full system — what might be considered the environmental, social, and governance factors of a system situated within a wider global system — is considered and deliberated upon. As such, the framework treats all digital health interventions as operating in a context where externalities must be accounted for (20).

Placing this framework alongside the wider literature clarifies both its contribution and its limits. A scoping review of digital health equity frameworks identified substantial convergence on the individual, interpersonal, community, and societal levels at which access barriers operate, but found that existing frameworks focus on social determinants and pay little attention to environmental burden or institutional accountability (45). The design-oriented review by Bucher et al. arrives at a comparable conclusion from a different direction, offering concrete equity tactics while remaining largely silent on environmental cost (18). Pierce argues explicitly that digital health equity cannot be sustained without attention to the material and ecological conditions of the technologies involved (12), and Coetzer et al. locate the deficit within health policy itself, finding that inclusive digital care requires policy instruments that most systems have not yet developed (13). At the global level, Tangcharoensathien et al. frame digital health as a health system function whose equitable and efficient operation depends on governance capacity rather than on technology adoption alone (4), a position consistent with the WHO global strategy (5). Read together, this body of work supports the interpretation advanced here: the social dimension of digital health is comparatively well theorised, the governance dimension is recognised but under-specified, and the environmental dimension remains largely external to the frameworks that decision-makers currently use. It is this asymmetry that the framework proposed below seeks to address.

### An integrated ESG framework for digital health

Building on the findings, this study proposes an ESG-informed framework for evaluating digital health innovations. This framework emphasizes the interdependence of environmental, social, and governance factors and supports a structured approach to decision-making. The framework's factors fall mainly in the social factor area but can be complemented by environmental and governance factors to provide an integrated view. Figure 1 demonstrates how incorporating environmental and governance dimensions can enhance the evaluation of digital health interventions and support more sustainable outcomes.

**Figure 1:**
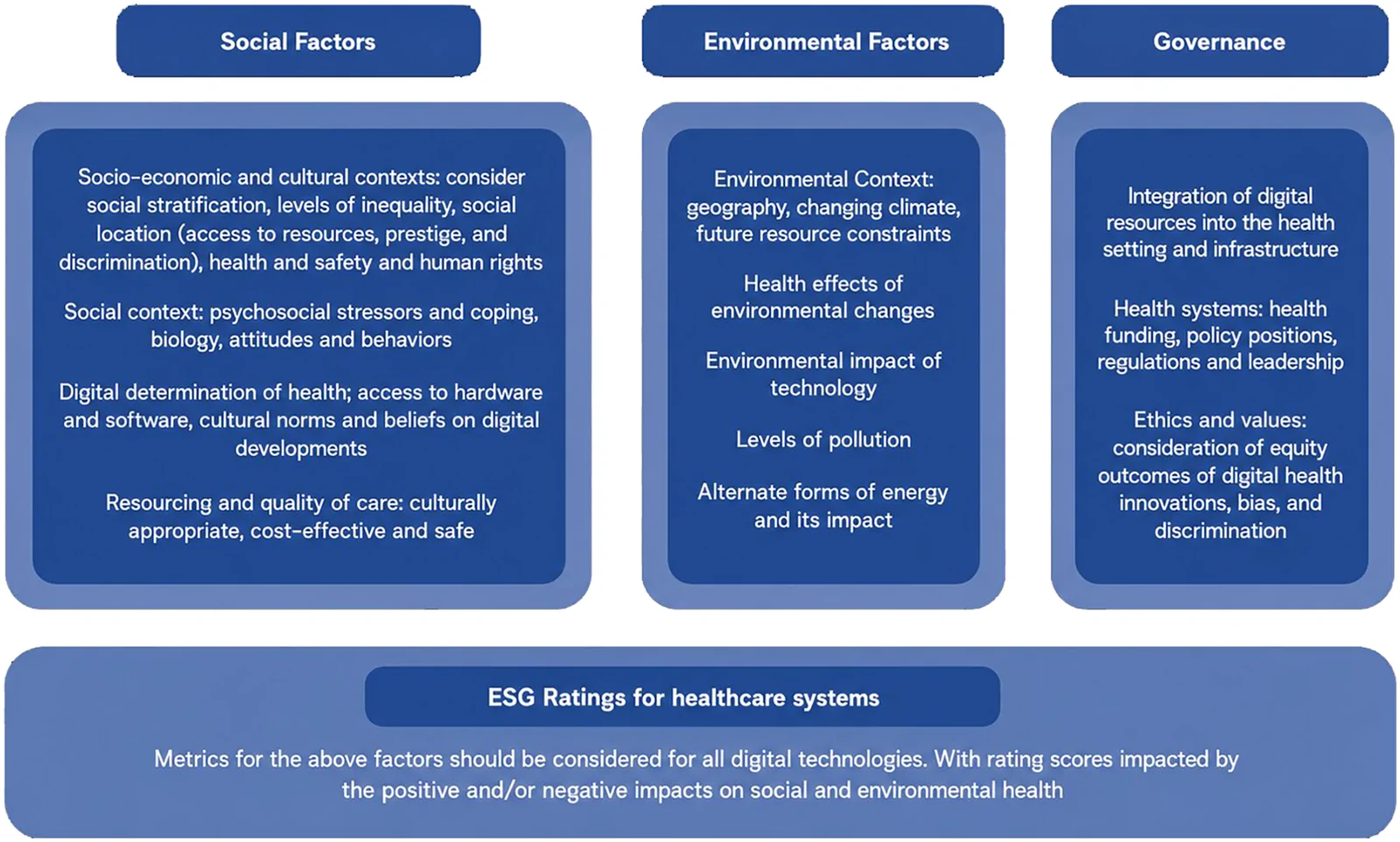
The digital health equity framework factors applied to ESG and digital health. Measurement of these factors should culminate in a framework capable of evaluating the positive and negative impacts of digital interventions on social and environmental health.

### An integration of the digital health equity model and ESG factors

Adaptation of the Digital Health Equity model to ESG decision-making for health innovations is described in Figure 2. This framework provides a basis for decision-making in the health innovation area for policymakers, healthcare providers, and development program implementers. Each element, as it is validated, should be assigned a rating of its potential positive or negative impact on social and environmental health. In this way, a valid assessment of impact in specific contexts may be made (4, 13, 45). While digital technologies are often viewed as solutions for environmental challenges, experts have cautioned that technological innovations alone cannot address climate change and should be supported by broader sustainability measures (46). In addition, the increasing accumulation of unused digital information, known as “dark data,” contributes to energy consumption and carbon emissions (47). Despite these concerns, digital health interventions such as telemedicine have been shown to reduce healthcare-related carbon footprints by minimizing patient and provider travel (44). Recognizing both the benefits and challenges of digitalization, the World Health Organization emphasizes the need for sustainable and responsible implementation of digital health technologies to support resilient healthcare systems (5).

**Figure 2:**
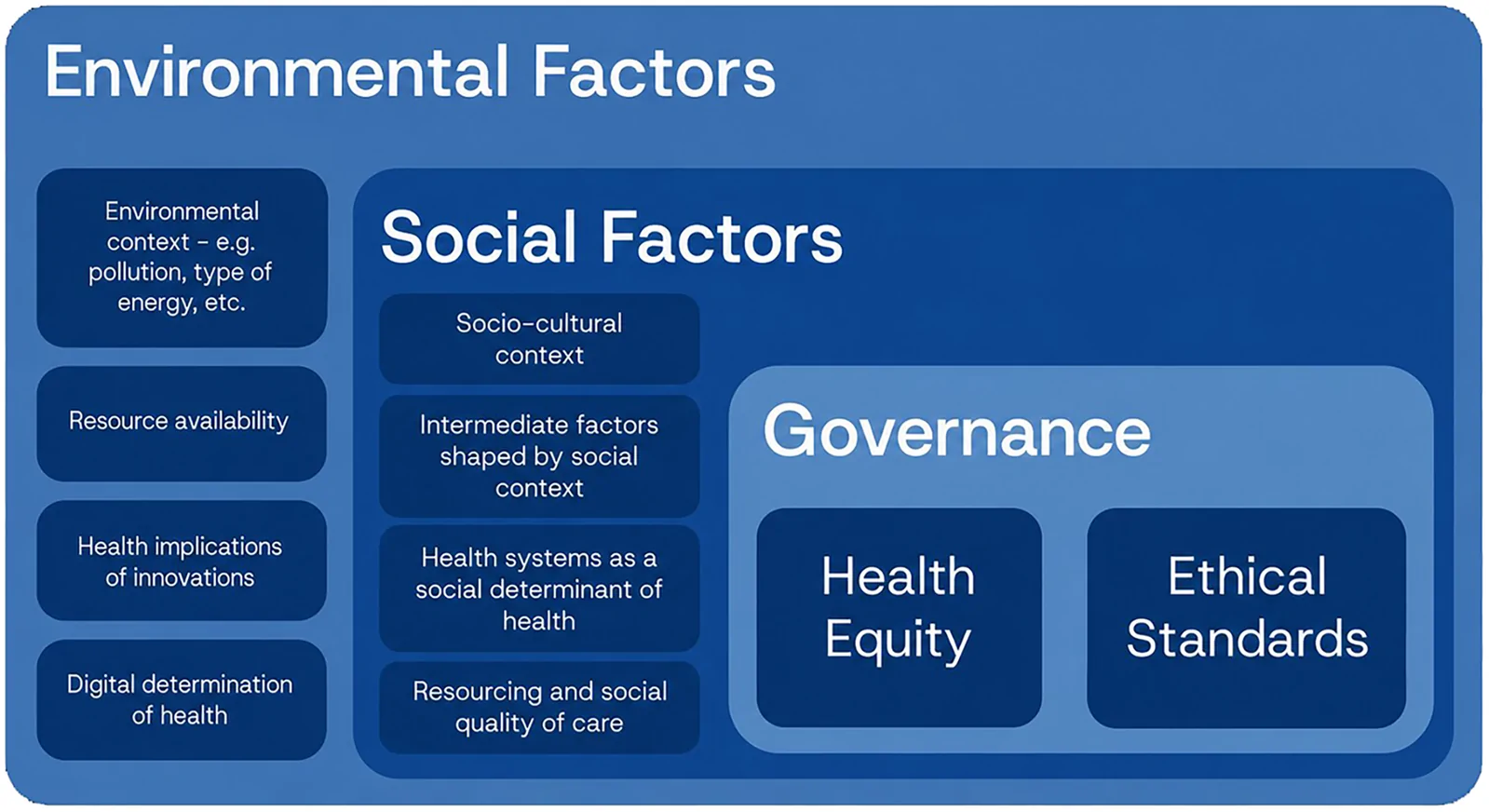
Adaptation of the digital health equity model to ESG decision-making for health innovations from Crawford and Serhal (20), licensed under CC BY 4.0.

### Operationalizing the framework: domain-level considerations

The framework treats ESG factors as integrated rather than parallel, on the premise that each domain conditions the others. It is accordingly not a measurement instrument but a conceptual model, intended to structure deliberation when digital health interventions are designed, procured, or evaluated. The considerations set out below indicate what each domain requires in practice; the question of metrics, weighting, and taxonomy is taken up under Future Directions.

At the environmental level, factors such as resource availability, scarcity, pollution, and the presence or absence of a circular economy should be considered. This level is broad because resources are limited and regulated, and they strongly influence national and institutional policies. It is important to assess the environmental impact of implementing or not implementing an innovation, including energy use and pollution. Digital health also fits here, as it involves hardware and software and overlaps with social factors such as digital literacy and integration into society.

At the social level, it is important to ensure that innovations are suitable for the socio-cultural context and to consider factors such as stress, beliefs, social norms, and resource availability. At the governance level, issues such as access, bias, geography, and inequality must be addressed. Ethical considerations should also be carefully evaluated for all interventions.

Governance factors within the framework include ethics in digital health — encompassing data sharing, privacy, AI bias, and responsible sourcing — as well as the alignment of health and environmental policy, which is particularly consequential when trade-offs and mitigation strategies must be managed (5, 7).

Environmental factors to consider include (23, 24):

- Geography and location: The global south is impacted more by climate change and is receiving fewer mitigation and adaptation resources. Consider also the direct climate impact of the area from extreme weather events and a changing climate, impacting food security and levels of poverty (32, 44).
- Environmental effects of digital hardware and software globally and locally from raw materials, toxic chemicals, use of energy, limited recyclability, to worker conditions.

Social considerations such as inherent bias, data integrity impact on privacy and data ownership (28), the social and human rights working conditions of technology workers and miners, and production areas that mine the essential minerals and develop and produce the technology are all considerations.

The lists and considerations are not exhaustive, but illustrative of how these factors must be considered to ensure ESG-related risks and opportunities are considered, and unintended consequences are reduced.

### Limitations

As a narrative synthesis, this review does not provide a systematic or exhaustive assessment of all available evidence and is subject to selection bias. Source identification was purposive and iterative, and screening counts were not maintained prospectively; consequently, the search is documented in outline but is not reproducible in the sense required of a systematic review, and no formal appraisal of methodological quality was undertaken. Evidence is also unevenly distributed across the three domains. Quantitative data on the environmental impacts of digital health specifically, as distinct from digital technologies in general, remain scarce, and governance evidence is fragmented across regulatory, ethical, and policy literatures that rarely address one another. Little primary evidence was identified from low- and middle-income countries, and the material discussed above in relation to those settings draws substantially on policy and intergovernmental sources rather than on empirical studies; conclusions concerning those contexts should be read accordingly. Finally, the proposed framework is conceptual and has not been validated in application. These constraints notwithstanding, the narrative design enables a broader, systems-oriented perspective, consistent with the aim of highlighting interdependencies, trade-offs, and the need for integrated ESG consideration in digital health innovation.

### Future directions

Four priorities follow from this review. First, metrics and ratings will need to be developed against each category in the model, together with a defensible basis for weighting them: assessing, for instance, the environmental impacts of mining, mineral extraction, transport, data processing, and energy source against the social impact of an AI-based document management system in a large hospital. At present there is no standardised approach to measuring ESG features in this domain, nor consensus on which metrics and indicators should be used, and a broad taxonomy of factors will be required over time. Second, that taxonomy and the framework itself require prospective validation in application, ideally alongside real procurement or implementation decisions, so that the framework's discriminating power can be tested rather than assumed. Third, empirical work is needed on the environmental footprint of specific digital health interventions, measured at the level of the deployment rather than the technology class, since it is at that level that trade-offs are actually incurred. Fourth, primary research from low- and middle-income settings is needed, both to establish whether the trade-offs identified here take the same form in those contexts and to ensure that the frameworks guiding global digital health investment are not derived exclusively from high-income evidence.

## Conclusion

This collaborative work from 10 international institutions provides a strong global foundation for considering ESG factors in digital health innovations and the need to consider their potential trade-offs. The inclusion of diverse global perspectives supports the development of such a framework, particularly when informed by the expertise and research of an international consortium, as in this research paper, to enhance the applicability of the framework across diverse settings. In this way, the full impact of digital health innovations can be evaluated, key considerations for their suitability across different contexts can be identified, and their global effects can be better understood.

At present, a comprehensive framework of ESG for digital health encompassing a detailed taxonomy, as well as standardized measures and indicators, does not yet exist. This remains an evolving and developing area, and the present research outlines foundational parameters to support its development. As digital health innovations continue to emerge and are rapidly adopted by health systems for clinical care, their potential to improve individual and community health and reduce health disparities underscores their importance in health and social development.

However, it is equally important to consider the balance between these innovations and their environmental and resource implications, including energy consumption, hardware use, and recyclability. As regulatory frameworks evolve, such considerations are likely to be incorporated into countries' strategic plans, as well as into cross-border taxes, for instance. Therefore, the adoption of digital health innovations must be carefully evaluated by weighing their benefits against potential environmental impacts, ethical considerations, and social implications, particularly regarding inequality. Digital health innovations are not neutral, and the SDGs cannot be effectively addressed, mitigated, or evaluated in isolation.

A framework such as the Digital Health Equity Framework, enhanced with ESG considerations, represents an important first step in supporting decision-makers, program implementers, and public health officials. Such an approach can promote balanced evaluation and provide an evidence-based foundation for decisions regarding the adoption and implementation of digital health innovations.

## Data Availability

The original contributions presented in the study are included in the article; further inquiries can be directed to the corresponding author.
